# Supplementary material for: Multiplex, high-throughput method to study cancer and immune cell mechanotransduction
Source: Commun Biol. 2024 Jun 1;7:674. doi: 10.1038/s42003-024-06327-x (PMC11144229; doi:10.1038/s42003-024-06327-x)
Supplement: Supplementary file 2 — Supplementary Information [file 42003_2024_6327_MOESM2_ESM.pdf]

Supplementary Information:

		STANDARD		LONG		WIDE BORE	
VIAFLO96 run speed	Flow rate (μL s^{-1})	t_f (s)	τ_{avg} (dyn cm^{-2})	t_f (s)	τ_{avg} (dyn cm^{-2})	t_f (s)	τ_{avg} (dyn cm^{-2})
1	11.80	14.38	0.1216	6.979	0.3519	18.55	0.06104
2	25.40	6.681	0.2618	3.242	0.7575	8.619	0.1314
3	63.20	2.685	0.6513	1.303	1.885	3.464	0.3269
4	80.70	2.103	0.8316	1.021	2.407	2.713	0.4174
5	98.30	1.726	1.013	0.8378	2.932	2.227	0.5085
6	129.1	1.314	1.330	0.6379	3.850	1.660	0.6678
7	185.9	0.9128	1.916	0.4430	5.544	1.178	0.9616
8	235.8	0.7196	2.430	0.3493	7.032	0.9284	1.220
9	265.3	0.6396	2.7340	0.3104	7.912	0.8252	1.372
10	294.8	0.5756	3.038	0.2794	8.792	0.7426	1.525

Table S1 The mean transit time and average fluid shear stress (FSS) that a cell experiences while traversing through the narrowing region of the "standard", "long" and "wide bore" INTEGRA pipette tips. These values were calculated using MATLAB at the ten relative operating speeds of the 300 μL VIAFLO96.

	30 G	22 G
Needle inner radius (cm)	7.94×10^{-3}	2.05×10^{2}
Volumetric flow (mL min^{-1})	14	18
Reynold's number	1850	914
Average shear stress (dyn cm^{-2})	3,950	290
Mean transit time (ms)	1.08	5.69

Table S2 Modified 22 G needle parameters. Poiseuille's equation was used to calculate the maximum FSS exposure, and the average FSS is equal to two thirds of the maximum FSS at each flow rate. The viscosity of RPMI media was assumed to be equal to water. Commonly used 30 G needle parameters are shown for reference[1,2].

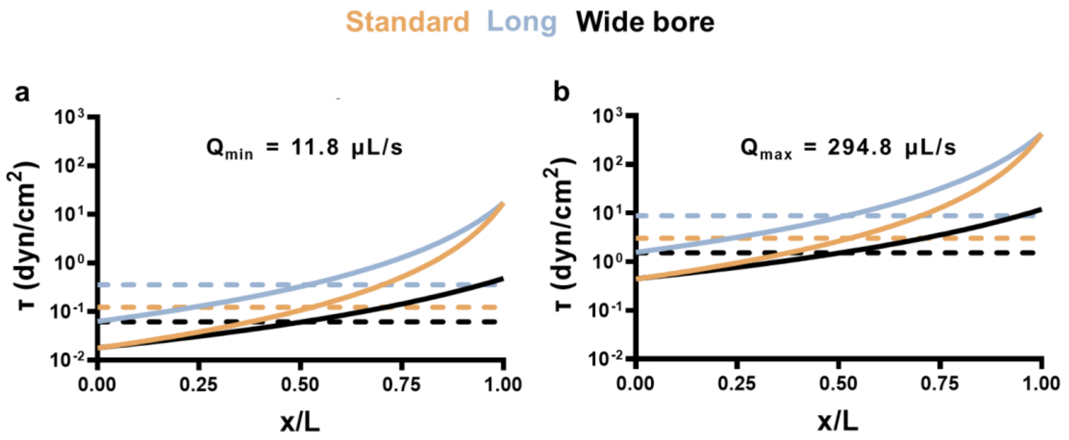

Fig. S1 Fluid shear stress analysis in the narrowing regions of the pipette tips. The time-averaged fluid shear stress (solid line) and average fluid shear stress (dashed line) estimated that a cell experiences traversing x/L in the various types of 300 μL INTEGRA pipette tips. Results shown for the (a) lowest and (b) highest VIAFLO96 operating speed. Determined using MATLAB.

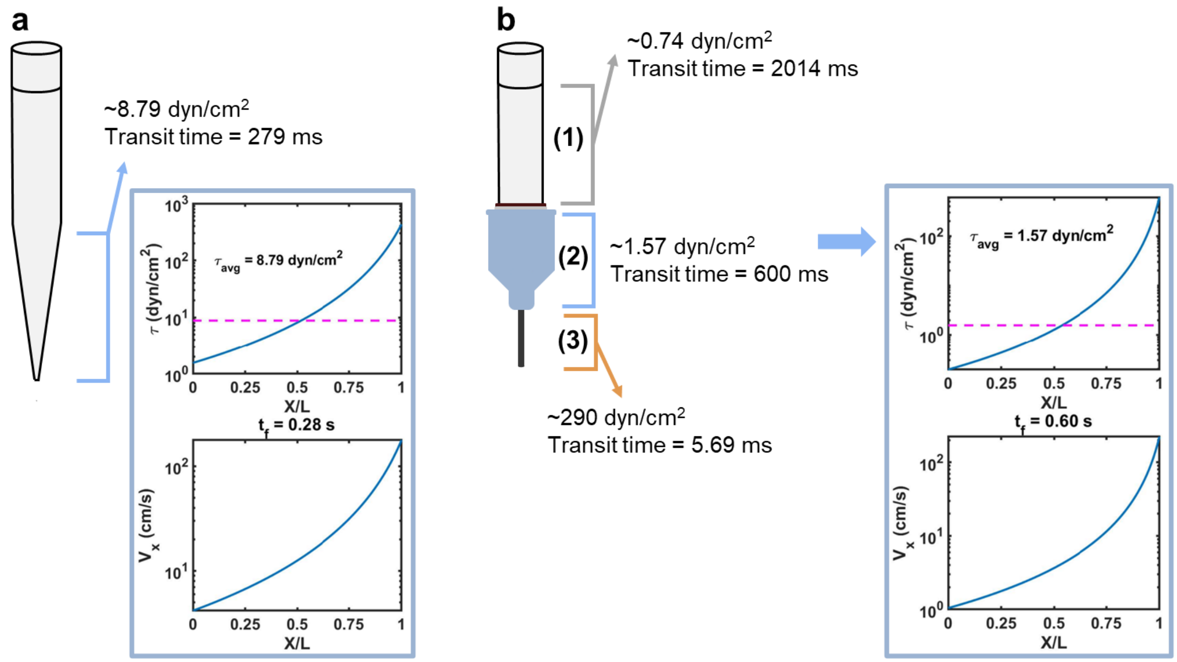

Fig. S2 Computational analysis of fluid shear stress and mean transit time in specific regions of the "long" INTEGRA pipette tips and 22 G needle. (a) Average FSS in narrowing region of "long" INTEGRA pipette tips. (b) Average FSS in the custom made 22 G needles in the (b1) upper region of the "long" tip, (b2) female luer region of the needle hub, and (b3) 22 G needle. (a) and (b2) are calculated using the narrowing region of a pipette tip model in MATLAB. (b1) and (b3) are assumed to have constant diameter, calculated using the Hagen-Poiseuille model of laminar fluid flow.

Mixing Cycles	Time (min)
500	20
1,000	40
2,000	80
5,000	200
10,000	400

Table S3 Approximate shearing durations of custom shearing programs using the VIAFLO96.

Number	Action	Summary
1	Move (X,Z)	X: -81.2 mm Z: 192.5 mm
2	Move Z	Z: 69.9 mm
3	Mix	25x 150.0 µL
4	Loop	Step: 3 Count: 19
5	Mix	25x 150.0 µL
6	Loop	Step: 5 Count: 19
7	Mix	25x 150.0 µL
8	Loop	Step: 7 Count: 19
9	Mix	25x 150.0 µL
10	Loop	Step: 7 Count: 19
11	Purge	Speed: 5
12	Move Z	Z: 192.5 mm

Table S4 Example VIAFLO96 shearing program developed using VIALINK to expose cells to 5,000 mixing cycles Script attached as a supplemental file *MECHANO5000.xml*.

VIAFLO96 run speed	Flow rate Q [µL s^{-1}]	Maximum FSS [dyn cm^{-2}]	Avg FSS [dyn cm^{-2}]
1	11.80	17.45	11.63
2	25.40	37.56	25.04
3	63.20	93.45	62.30
4	80.70	119.3	79.55
5	98.30	145.4	96.90
6	129.1	190.9	127.3
7	185.9	274.9	183.3
8	235.8	348.7	232.5
9	265.3	392.3	261.5
10	294.8	435.9	290.6

Table S5 The average fluid shear stress that a cell experiences while traveling through a 22 G needle. Calculated at speed settings of 1–10 of the VIAFLO96. Determined using Poiseuille's equation, assuming the viscosity of RPMI media to be equal to water, with an inner needle radius of 2.05 x 10^{-2} cm.

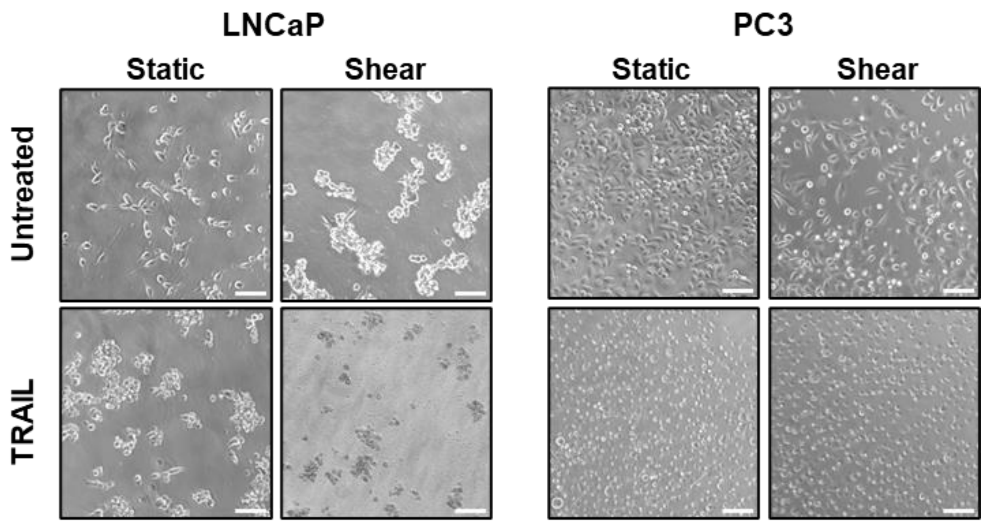

Fig. S3 Representative brightfield images of prostate cancer cells post-fluid shear stress exposure. Images depict prostate cancer cells after exposure to 5,000 shearing cycles using the VIAFLO96 (22 G needles) with TRAIL treatment. Scale bar = 100 µm.

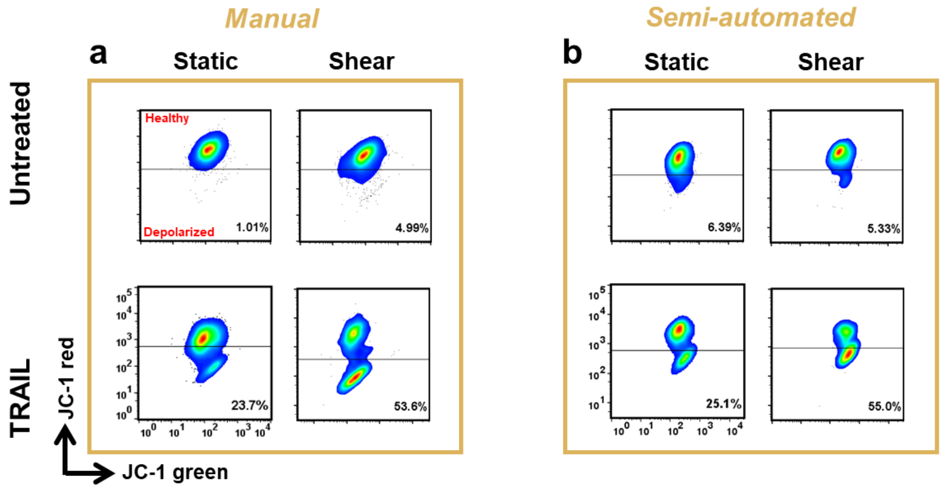

Fig. S4 Manual and semi-automated depolarized mitochondria results. Representative JC-1 flow cytometry plots for PC3 cells exposed to 5,000 shearing cycles using the VIAFLO96 (22 G needles) with TRAIL treatment for the (a) manual and (b) semi-automated staining methods.

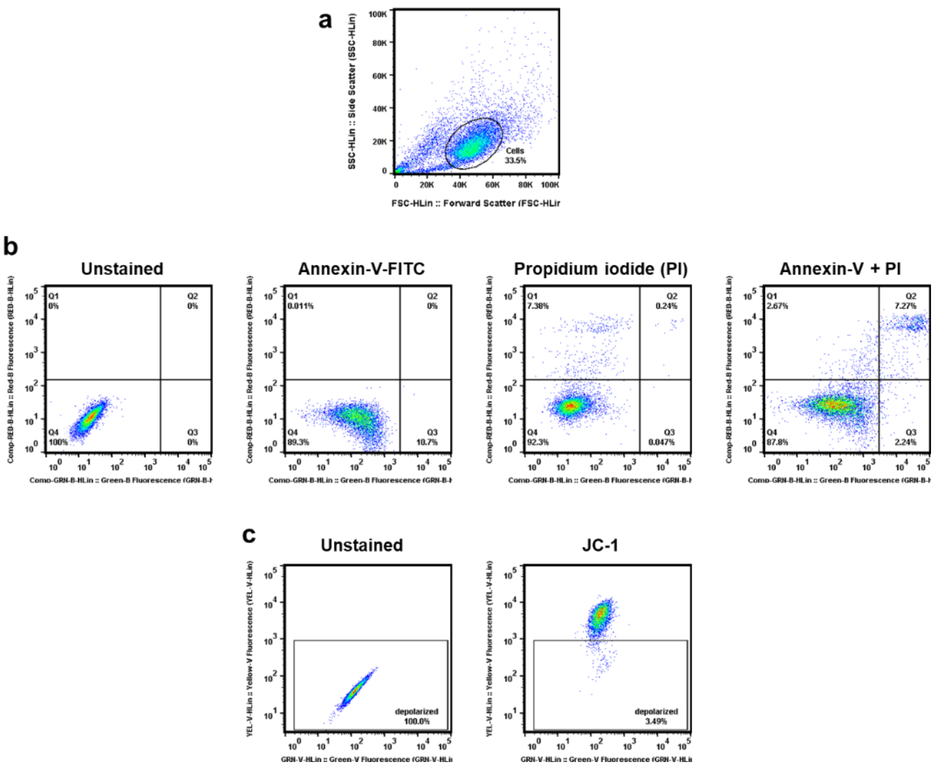

Fig. S5 Representative flow cytometry gating strategy for prostate cancer studies. (a) Selection of cancer cell population from the SSC vs. FSC flow plots. (b) Staining controls used for each AV/PI assay and (c) unstained control used to confirm gating of the JC-1 assay.

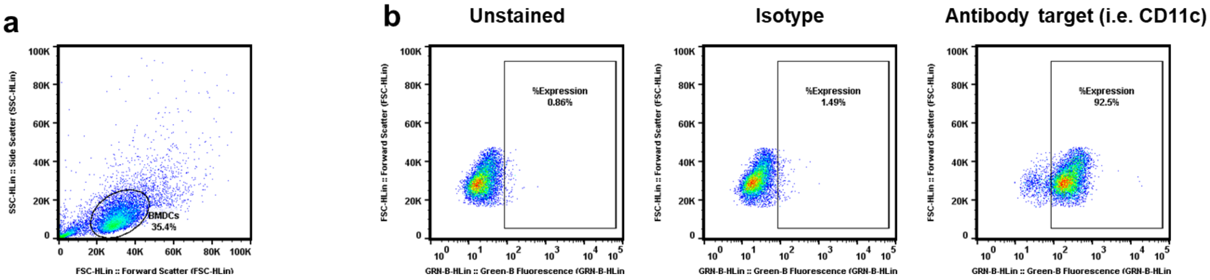

Fig. S6 Representative flow cytometry gating strategy for the bone marrow dendritic cell (BMDC) analysis. (a) Selection of BMDC population from the SSC vs. FSC flow plots. (b) Unstained and isotype controls to gate the target of interest.

Supplementary References:

1. Barnes, J. M., Nauseef, J. T. & Henry, M. D. Resistance to Fluid Shear Stress Is a Conserved Biophysical Property of Malignant Cells. *PLoS One* **7**, e50973 (2012).
2. Hope, J. M. *et al.* Circulating prostate cancer cells have differential resistance to fluid shear stress-induced cell death. *J Cell Sci* **134**, jcs251470 (2021).
