# Supplementary material for: Multiplex, high-throughput method to study cancer and immune cell mechanotransduction
Source: Commun Biol. 2024 Jun 1;7:674. doi: 10.1038/s42003-024-06327-x (PMC11144229; doi:10.1038/s42003-024-06327-x)
Supplement: Supplementary file 3 — Description of Additional Supplementary Files [file 42003_2024_6327_MOESM3_ESM.pdf]

Description of Additional Supplementary Files

File name: Supplementary Data 1
Description: Detailed statistical analysis from the manuscript figures.

File name: Supplementary Data 2
Description: The source data for the figures in the manuscript.
