# Supplementary material for: Multiplex, high-throughput method to study cancer and immune cell mechanotransduction
Source: Commun Biol. 2024 Jun 1;7:674. doi: 10.1038/s42003-024-06327-x (PMC11144229; doi:10.1038/s42003-024-06327-x)
Supplement: Supplementary file 8 — Reporting Summary [file 42003_2024_6327_MOESM8_ESM.pdf]

nature portfolio

Corresponding author(s): King, Michael R.

Last updated by author(s): 5/9/2024

# Reporting Summary

Nature Portfolio wishes to improve the reproducibility of the work that we publish. This form provides structure for consistency and transparency in reporting. For further information on Nature Portfolio policies, see our Editorial Policies and the Editorial Policy Checklist.

## Statistics

For all statistical analyses, confirm that the following items are present in the figure legend, table legend, main text, or Methods section.

| n/a | Confirmed | |
|---|---|---|
| ☐ | ☒ | The exact sample size (*n*) for each experimental group/condition, given as a discrete number and unit of measurement |
| ☐ | ☒ | A statement on whether measurements were taken from distinct samples or whether the same sample was measured repeatedly |
| ☐ | ☒ | The statistical test(s) used AND whether they are one- or two-sided<br>*Only common tests should be described solely by name; describe more complex techniques in the Methods section.* |
| ☒ | ☐ | A description of all covariates tested |
| ☐ | ☒ | A description of any assumptions or corrections, such as tests of normality and adjustment for multiple comparisons |
| ☐ | ☒ | A full description of the statistical parameters including central tendency (e.g. means) or other basic estimates (e.g. regression coefficient) AND variation (e.g. standard deviation) or associated estimates of uncertainty (e.g. confidence intervals) |
| ☐ | ☒ | For null hypothesis testing, the test statistic (e.g. *F*, *t*, *r*) with confidence intervals, effect sizes, degrees of freedom and *P* value noted<br>*Give P values as exact values whenever suitable.* |
| ☒ | ☐ | For Bayesian analysis, information on the choice of priors and Markov chain Monte Carlo settings |
| ☒ | ☐ | For hierarchical and complex designs, identification of the appropriate level for tests and full reporting of outcomes |
| ☒ | ☐ | Estimates of effect sizes (e.g. Cohen's *d*, Pearson's *r*), indicating how they were calculated |

*Our web collection on statistics for biologists contains articles on many of the points above.*

## Software and code

Policy information about availability of computer code

| | |
|---|---|
| Data collection | N/A |
| Data analysis | Data sets were plotted and analyzed for statistical analysis using Prism 10 software (GraphPad, San Diego, CA, USA). |

For manuscripts utilizing custom algorithms or software that are central to the research but not yet described in published literature, software must be made available to editors and reviewers. We strongly encourage code deposition in a community repository (e.g. GitHub). See the Nature Portfolio guidelines for submitting code & software for further information.

## Data

Policy information about availability of data

All manuscripts must include a data availability statement. This statement should provide the following information, where applicable:

- Accession codes, unique identifiers, or web links for publicly available datasets
- A description of any restrictions on data availability
- For clinical datasets or third party data, please ensure that the statement adheres to our policy

The data that support the findings of this study are available within the article and its supplementary material.

## Research involving human participants, their data, or biological material

Policy information about studies with human participants or human data. See also policy information about sex, gender (identity/presentation), and sexual orientation and race, ethnicity and racism.

| | |
|---|---|
| Reporting on sex and gender | N/A |
| Reporting on race, ethnicity, or other socially relevant groupings | N/A |
| Population characteristics | N/A |
| Recruitment | N/A |
| Ethics oversight | N/A |

Note that full information on the approval of the study protocol must also be provided in the manuscript.

# Field-specific reporting

Please select the one below that is the best fit for your research. If you are not sure, read the appropriate sections before making your selection.

☒ Life sciences ☐ Behavioural & social sciences ☐ Ecological, evolutionary & environmental sciences

For a reference copy of the document with all sections, see nature.com/documents/nr-reporting-summary-flat.pdf

# Life sciences study design

All studies must disclose on these points even when the disclosure is negative.

| | |
|---|---|
| Sample size | No sample size calculation performed. |
| Data exclusions | N/A |
| Replication | At least three independent replicates were used for each experiment. |
| Randomization | N/A |
| Blinding | N/A |

# Reporting for specific materials, systems and methods

We require information from authors about some types of materials, experimental systems and methods used in many studies. Here, indicate whether each material, system or method listed is relevant to your study. If you are not sure if a list item applies to your research, read the appropriate section before selecting a response.

## Materials & experimental systems

| n/a | Involved in the study | |
|---|---|---|
| ☐ | ☒ | Antibodies |
| ☐ | ☒ | Eukaryotic cell lines |
| ☒ | ☐ | Palaeontology and archaeology |
| ☐ | ☒ | Animals and other organisms |
| ☒ | ☐ | Clinical data |
| ☒ | ☐ | Dual use research of concern |
| ☒ | ☐ | Plants |

## Methods

| n/a | Involved in the study | |
|---|---|---|
| ☒ | ☐ | ChIP-seq |
| ☐ | ☒ | Flow cytometry |
| ☒ | ☐ | MRI-based neuroimaging |

## Antibodies

| | |
|---|---|
| Antibodies used | Propidium iodide staining solution (BD Pharmingen, Cat# 556463)<br>FITC-conjugated Annexin-V antibody (BD Pharmingen, Cat# 556419)<br>JC-1 mitochondrial membrane potential dye (Abcam, Cat# ab141387)<br>FITC-conjugated anti-mouse CD11c antibody (Clone: N418, BioLegend, Cat# 117306) |

| | |
|---|---|
| | PE-conjugated anti-mouse I-A/I-E (MHC II) antibody (Clone: M5/114.15.2, BioLegend, Cat# 107608)<br>PE-conjugated phospho-NF-kB p65 (Ser529) antibody (Clone: NFkBp65S529-H3, Thermo Scientific, Cat# MA5-37165)<br>PE-conjugated mouse anti-Ki67 antibody (Clone: B56, BD Pharmingen, Cat# 567719)<br>PE-conjugated anti-mouse CD80 antibody (Clone: 16-10A1, BD Pharmingen, Cat# 553769) |
| Validation | All antibodies were validated by their manufacturers and were used according to the manufacturer's directions. When necessary, corresponding isotypes were included in experiments to enhance gating accuracy. |

## Eukaryotic cell lines

Policy information about cell lines and Sex and Gender in Research

| | |
|---|---|
| Cell line source(s) | LNCaP & PC3 immortalized cell lines, derived from human males (ATCC).<br>Bone marrow-derived dendritic cells (BMDCs) isolated from the femurs of healthy female, eight-week-old BALB/c mice. |
| Authentication | The LNCaP cell lines were authenticated in March 2023. The PC3 cell line was not, but both cell lines were ordered from ATCC. The BMDCs were treated with GM-CSF following isolation to generate and promote DC differentiation of precursor DCs and unconventional precursor DCs. |
| Mycoplasma contamination | Cell lines were not tested for mycoplasma contamination during the courses of these experiments. |
| Commonly misidentified lines<br>(See ICLAC register) | We did not use misidentified cell lines in this study. |

## Animals and other research organisms

Policy information about studies involving animals; ARRIVE guidelines recommended for reporting animal research, and Sex and Gender in Research

| | |
|---|---|
| Laboratory animals | Healthy female BALB/c mice were used for these studies, 6 - 8 weeks old (Jackson Laboratories). |
| Wild animals | Wild animals were not used in this study. |
| Reporting on sex | No sex report on this study. |
| Field-collected samples | No samples were collected from the field in this study. |
| Ethics oversight | All animal studies were approved by the Vanderbilt IACUC Protocol #M1700009-02 and mice were monitored by staff from the Division of Animal Care (DAC) at Vanderbilt University. |

Note that full information on the approval of the study protocol must also be provided in the manuscript.

## Plants

| | |
|---|---|
| Seed stocks | N/A |
| Novel plant genotypes | N/A |
| Authentication | N/A |

## Flow Cytometry

### Plots

Confirm that:

- [x] The axis labels state the marker and fluorochrome used (e.g. CD4-FITC).
- [x] The axis scales are clearly visible. Include numbers along axes only for bottom left plot of group (a 'group' is an analysis of identical markers).
- [x] All plots are contour plots with outliers or pseudocolor plots.
- [x] A numerical value for number of cells or percentage (with statistics) is provided.

## Methodology

| | |
|---|---|
| Sample preparation | All cells for all experiments were stained with antibodies following exposure to fluid shear stress.<br><br>Live cell viability staining: Cancer cells were washed in buffer and stained with appropriate dilutions of PI/ Annexin-V for 15 min in the dark.<br><br>JC1 staining: Cancer cells were washed in buffer, stained with the appropriate dilution of the JC-1 dye for 30 min in the dark at 37 °C, then washed again.<br><br>Intracellular staining BMDCs: BMDCs were fixed with 4% paraformaldehyde and permeabilized in 100% ice-cold methanol. Samples were incubated with antibodies in the dark (CD11c, MHC II, phospho-NF-kB, Ki67 and CD80), and excess antibody was washed out of solution prior to flow cytometry analysis.<br><br>Staining for the "semi-automated" cancer cell experiments and all BMDC experiments was completed using the Viaflo96 device. |
| Instrument | Guava easyCyte 12HT benchtop flow cytometer (MilliporeSigma). |
| Software | All flow cytometry software was analyzed using FlowJo software (version 10). |
| Cell population abundance | Gating for pure cancer cell or BMDC populations for the flow cytometry analysis is shown in Supplemental Figures S5 and S6, completed using FlowJo. When experiments were run in the flow cytometer, each well (sample) was set to collect 5,000 events at a minimum. |
| Gating strategy | The gating strategy for the experiments is shown in Figures S5 and S6. The initial cell gating selected populations of cancer cells or BMDCs to exclude cell debris due to fluid shear stress exposure. For the Annexin-V/ PI cell viability assay, the staining controls were used to first perform compensation between the red-b (PI) and green-b (Annexin-V-FITC) lasers. |

☒ Tick this box to confirm that a figure exemplifying the gating strategy is provided in the Supplementary Information.
